# Giant oscillating thermopower at oxide interfaces

**DOI:** 10.1038/ncomms7678

**Published:** 2015-03-27

**Authors:** Ilaria Pallecchi, Francesca Telesio, Danfeng Li, Alexandre Fête, Stefano Gariglio, Jean-Marc Triscone, Alessio Filippetti, Pietro Delugas, Vincenzo Fiorentini, Daniele Marré

**Affiliations:** 1 Department of Physics, CNR-SPIN and Genova University, via Dodecaneso 33, Genova 16146, Italy; 2 Department of Quantum Matter Physics,, University of Geneva, 24 Quai E.-Ansermet, Geneva 4 1211, Switzerland; 3 CNR-IOM UOS Cagliari, c/o Dipartimento di Fisica, Università di Cagliari, S.P. Monserrato-Sestu Km.0,700, Monserrato (Ca) 09042, Italy; 4 CompuNet, Istituto Italiano di Tecnologia—IIT, Via Morego 30, Genova 16163, Italy; 5 Dipartimento di Fisica, Università di Cagliari, CNR-IOM, S.P. Monserrato-Sestu Km.0,700, Monserrato (Ca) 09042, Italy

## Abstract

Understanding the nature of charge carriers at the LaAlO_3_/SrTiO_3_ interface is one of the major open issues in the full comprehension of the charge confinement phenomenon in oxide heterostructures. Here, we investigate thermopower to study the electronic structure in LaAlO_3_/SrTiO_3_ at low temperature as a function of gate field. In particular, under large negative gate voltage, corresponding to the strongly depleted charge density regime, thermopower displays high negative values of the order of 10^4^–10^5^μVK^−1^, oscillating at regular intervals as a function of the gate voltage. The huge thermopower magnitude can be attributed to the phonon-drag contribution, while the oscillations map the progressive depletion and the Fermi level descent across a dense array of localized states lying at the bottom of the Ti 3d conduction band. This study provides direct evidence of a localized Anderson tail in the two-dimensional electron liquid at the LaAlO_3_/SrTiO_3_ interface.

## Abstract (web summary)

Electrons sitting in quantum wells at the interface between two oxides can localize in the disordered potential, completely changing their properties. Here, the authors observe unexpectedly large thermopower that oscillates upon carrier density modulation and ascribe it to strong electron-phonon coupling.

## Graphical abstract

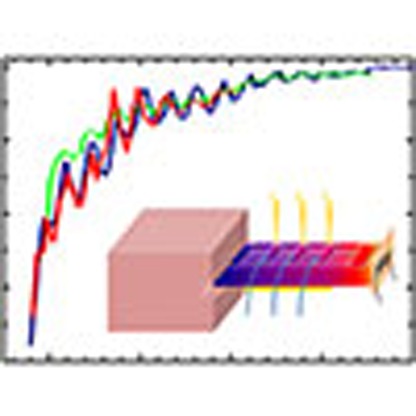

The electronic properties of the two-dimensional (2D) electron system found at the LaAlO_3_/SrTiO_3_ (LAO/STO) interface^1^, characterized by Hall-measured sheet carrier densities *n*_2D_*~*2–8 × 10^13^ cm^−2^ and a thickness of a few nm, are deeply affected by the electronic confinement, which leads to an anisotropic spatial extension of Ti *3d* conduction bands and to a sub-band structure of the *t*_*2g*_ levels. This geometry favours the spontaneous charge localization within *d*_*xy*_ levels spatially very confined (≅2 nm) on the SrTiO_3_ (STO) side of the interface. This electronic confinement and breaking of inversion symmetry is also at the origin of a strong Rashba spin-orbit coupling^2^.

When the electronic density is large enough, Ti *3d*_*xz*_−*d*_*yz*_ sub-bands with a larger spatial extension are progressively filled with consequences on magnetotransport and possibly on superconductivity^3^,^4^. Less well understood is the nature of the low-density regime on the verge of localization, a key aspect for field-effect applications. Experiments have shown that a sharp transition to a highly resistive state occurs at a critical density of 0.5–1.5 × 10^13^ cm^−2^ (ref. 5). Considerations based on the polarization catastrophe model^6^ suggest that a large portion of the electron charge present at the interface may actually be trapped in localized levels located in energy somewhere below the band mobility edge. The presence of electrons localized by impurities, disorder or polaronic behaviour, has been reported in a number of works^7^,^8^,^9^,^10^. So far, however, only indirect evidences of these localized states were furnished, based on features in optical and transport properties.

Field-effect is unquestionably the most powerful approach to study the fundamental properties of oxide heterostructures at varying charge density. Under gate voltage, the phase diagram can be cleanly and reversibly explored without the complications inherent to chemical doping, which may easily result in additional effects blurring the intrinsic behaviour of these systems. For the LAO/STO system, field-effect has been extensively used to reveal tunable superconductivity^11^,^12^, strong Rashba spin-orbit^2^,^3^, enhanced capacitance and negative compressibility^13^, and magnetic effects^14^,^15^,^16^,^17^. Several works^5^,^13^,^18^,^19^ in particular have emphasized the correlated/localized nature of the electron carriers at large negative gate voltage, corresponding to a strongly charge-depleted regime characterized by sheet resistance of the order MΩ or higher, and densities lower than *n*_2D_*~*10^13^ cm^−2^.

Among transport measurements, the most direct probe of the electronic properties of a metal is certainly furnished by thermopower (or Seebeck coefficient, *S*). In contrast with magnetoresistivity (that is, Shubnikov de Haas) measurements^20^,^21^, which require high-mobility samples and strong magnetic fields, *S* can be equally well measured at low and high temperature and for the whole range of Hall-measurable charge densities. Specifically, *S* is the sum of two terms, the diffusive Seebeck (*S*_d_), which is the potential drop generated by electrons diffused by a thermal gradient, and the phonon-drag Seebeck (*S*_g_), that is, the additional electron current generated by the coupling of electrons with diffused phonons. *S*_d_, related to the energy derivative of the conductivity^22^, is much less crucially dependent on the scattering regime than conductivity or mobility, and represents a very sensitive probe of the electronic density of states (DOS) of the system. On the other hand, *S*_g_ is directly related to the electron-phonon coupling (EPC), and may be used as a probe for a quantitative evaluation of the momentum relaxation rate of the electrons due to EPC^23^.

Note that very large values of the Seebeck coefficient have been measured in titanium oxides, even in the shape of three-dimensional samples (see, for example, refs 24, 25). More recently, Ohta and co-workers^26^,^27^,^28^ have studied the enhancement of the Seebeck effect driven by charge confinement in titanate heterostructures, both in the diffusion and drag regimes.

In this work, we perform Seebeck measurements under field effect, focusing on the depletion regime. We find that *S* assumes huge negative values and oscillates while the Fermi level is progressively lowered. We consider this behaviour as an evidence of charge localization below the bottom of the conduction band.

## Results

### Transport and thermopower measurements

**Figure 1:**
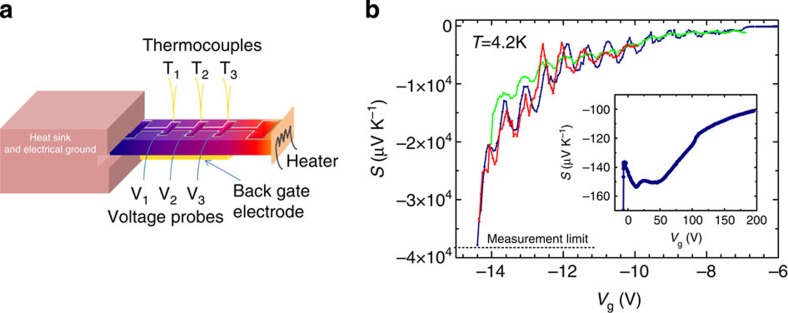
Seebeck measurement configuration and behaviour under gate field of a LAO/STO interface. (**a**) Sketch of the sample and experimental configuration for the Seebeck measurements: the two-dimensional electron liquid lies in the (001) plane of the SrTiO_3_, whereas the thermal gradient is applied along the 010 direction. (**b**) Seebeck coefficient versus gate voltage measured in a LAO/STO interface at 4.2 K. In the main panel, the different traces correspond to different thermal and *V*_g_ cycles: the red curve is measured using an *ac* heat flow whose power is 3.5 times smaller than the one (~mW) used for the blue and green curves. The blue and red curves are measured with decreasing gate voltage, whereas the green curve is measured with increasing gate voltage. The measurement limit related to the finite input impedance of the instruments used for the measurement of the voltage is also indicated. In the inset, a blow-up of the accumulation regime (*V*_g_>0) is shown.

Transport and thermopower under applied gate voltage of several LAO/STO samples are measured. As all the samples show similar results, only one of them will be presented in the following (the others are described in the Supplementary Note 1 and shown in Supplementary Fig. 1). Seebeck effect is measured in a home-made cryostat, from 4.2 K to room temperature, using an ac technique^29^, in the configuration sketched in Fig. 1a. Hall effect and resistivity data are measured in a Quantum Design Physical Properties Measurement System (PPMS), from 2 K to room temperature and in magnetic field up to 9 T. Both dc and ac techniques are used. In particular, in the depletion regime, the ac lock-in technique is preferable, in that it allows very low currents (~10 nA) to be applied and thus pinch-off problems to be avoided.

In Fig. 1b, the Seebeck coefficient measured at 4.2 K as a function of the gate voltage *V*_g_ is displayed, for two different thermal cycles. *S* is negative at any voltage, indicating electron-like carriers. For positive *V*_g_ (see inset), which scans the charge accumulation regime, *S* shows the magnitude (~100 μV K^−1^) typically seen in STO bulk or heterostructures^30^,^31^,^32^ and varies by no more than 60% across the whole positive range, exhibiting a non-monotonic behaviour. In this accumulation regime, the measured magnitude of *S* first displays a very small increment up to *V*_g_~20 V, and then it decreases regularly at larger *V*_g_; assuming diffusive regime, the lowering of *S* is simply a consequence of the Fermi energy increase occurring along with the progressive charge accumulation. If one looks now at the negative *V*_g_ region, after a featureless gate voltage interval of ~7 V, something really astonishing happens: *S* progressively bursts to high values of the order of ~10^4^ μV K^−1^ (it changes from *S*=−80 μV K^−1^ at *V*_g_=−5 V to −26,000 μV K^−1^, that is more than two orders of magnitude, for a *V*_g_ variation of only 10 V). Furthermore, *S* dramatically oscillates with *V*_g_. These oscillations are regularly distributed, and roughly occur at voltage intervals of 0.6 V. Above a maximum limit of *V*_g_~ −15 V, no thermoelectric voltage signal can be detected experimentally anymore, the sample entering a highly insulating state. We may argue, however, that in principle the exponential rise of *S* to even greater negative values and the associated oscillations could be continued up to the ideal limit of complete charge depletion (in the Supplementary Note 1, a sample with *S* reaching ~−10^5^ μV K^−1^ is shown as well). It is interesting to note that slight shifts of the *S* curves are observed after different cycles of temperature and *V*_g_, as explained in the Methods and Supplementary Note 1 sections, however, the oscillating and diverging behaviour is always present. In particular, oscillating and diverging *S* curves are systematically observed at low negative *V*_g_ values after a forming protocol of *V*_g_ cycle at low temperature. The protocol is to apply at low temperature first the highest positive voltage *V*_max_ to be used and then to sweep the voltage between *V*_max_ and *V*_min_. This procedure allows reproducible measurements to be obtained.

**Figure 2:**
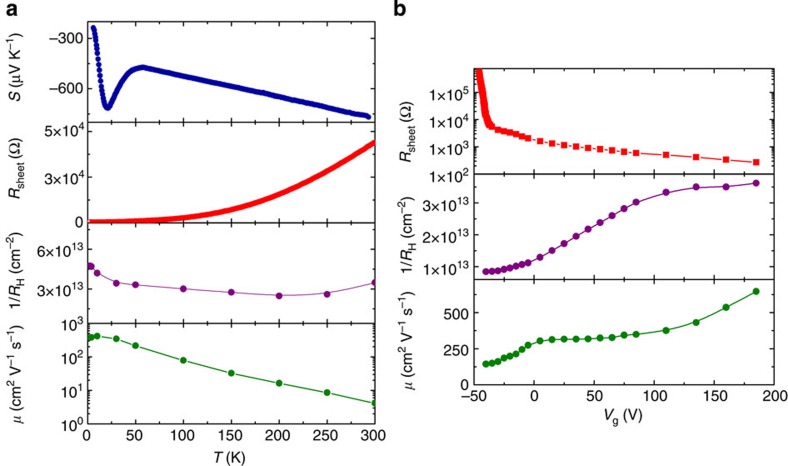
Electric and thermoelectric properties of LAO/STO interfaces. (**a**) Electric and thermoelectric properties of the two-dimensional electron liquid as a function of temperature. From top to bottom are Seebeck coefficient, sheet resistance, inverse Hall constant and Hall mobility. (**b**) Electric transport properties of the LAO/STO interface at 4.2 K as a function of the gate voltage, namely sheet resistance, inverse Hall constant and carrier Hall mobility.

In Fig. 2a,b, the characterization of electric and thermoelectric properties as a function of *T* (at *V*_g_=0) and *V*_g_ (at *T*=4.2 K) is reported, respectively. From the measurement of longitudinal and Hall resistances, sheet carrier density *n*_2D_ (which as usual we identify with the inverse Hall resistance assuming the Hall factor equal to unity and single band description) and Hall mobility *μ* are extracted. As a function of *T* and at zero gate voltage, the sample shows features typical of standard LAO/STO interfaces^30^: *S* exhibits a phonon-drag peak at low temperature and is linear above 50 K, as expected in the diffusive regime. *R*_sheet_ decreases with decreasing *T*, as expected for phonon-limited metallic behaviour, and saturates at a residual value of 700 Ω. The sheet carrier density *n*_2D_ extracted from the inverse Hall constant varies, depending on the samples, in the range 3–8 × 10^13^ cm^−2^ as is common for LAO/STO samples. A slight *R*_H_ downturn at low temperature has also been observed in other similar samples^33^ and may be interpreted in terms of charge delocalization related to the increased screening at low temperature, where the dielectric permittivity is very large. Mobility has a low-*T* value around 380 cm^2^ V^−1^ s^−1^ and decreases approximately as *T*^−2^ at high *T*, as expected from scattering by optical phonons. The same properties measured at fixed *T*=4.2 K and varying *V*_g_ (Fig. 2b) depict an accumulation-depletion scenario, with *R*_sheet_ and *n*_2D_ being decreasing and increasing functions of *V*_g_, respectively. *R*_sheet_ shows a sharp change in slope at *V*_g_=0, that is at the border of accumulation and depletion regions, similar to what was reported in previous field-effect measurements at low *T*^12^,^18^, and reaches values of the order of 10^4^–10^5^ Ω for *V*_g_*<*−40 V corresponding to a maximally depleted sheet density *n*_2D_~8 × 10^12^ cm^−2^. Below this threshold, *R*_sheet_ cannot be measured any longer since a sharp metal-insulator transition occurs. Consistently, we find the Hall mobility to decrease with negative *V*_g_ up to the lowest measurable value, in agreement with the literature^19^.

## Discussion

From the measurements two essential concepts emerge: (i) on the positive side of the gate field, transport properties are compatible with ordinary 2D electron gas (2DEG) behaviour in the charge accumulation regime; a switch towards negative gate values drags the system towards a highly depleted regime characterized by a sharp rise of resistivity and decrease of mobility, prefiguring a transition from metallic to localized charge transport behaviour; (ii) At low *T* this progressive depletion is indicated by rapidly diverging and dramatically oscillating Seebeck coefficient. In the absence of any magnetic field, this huge oscillating thermopower has no exact precedent in the literature, to our knowledge. In a small number of works a large field effect enhancement of the Seebeck coefficient is reported. The closest analogies with our result are probably the diverging thermopower and electrical resistivity measured in Silicon 2DEG^34^, occurring near the metal-insulator transition and attributed to Anderson localization, and the oscillating thermopower as a function of the carrier concentration found in GaAs/AlGaAs 2DEGs^35^ and in single-layer MoS_2_ (ref. 36), attributed, respectively, to electron-localization phenomena and variable range hopping mechanism. In both cases, however, the measured *S* coefficients (~10^3^ μV K^−1^ for Si and ~10^2^ μV K^−1^ for GaAs 2DEG) remain well below our values, and in both papers the dominant thermoelectric contribution is attributed to electron diffusion. Although we agree that electron localization must be the appropriate electronic structure landscape for this phenomenon, our standing point is that the measured thermopower is incompatible with electron diffusion, and must be primarily due to phonon drag, which is known to dominate over diffusion in certain ranges of temperature and densities where the EPC is strong.

Evidence of large EPC in STO bulk and STO-based heterostructures related to electron localization has been reported in several works^7^,^8^,^37^,^38^. EPC can ignite large phonon drag according to the following expression for *S*_*g*_ in the *j* direction (the detailed formulation^39^,^40^,^41^ is reported in the Supplementary Notes 4–7):


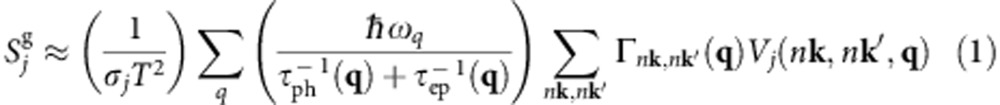


where *σ*_*j*_ is the electron conductivity, *V*_*j*_ a velocity factor, Γ_*n***k***,n***k′**_ the electron-phonon scattering matrix, *τ*_ep_ the phonon relaxation time due to EPC, whereas *τ*_ph_ sums up all the other relevant relaxation mechanisms (for example, phonon–phonon, phonon–boundary, phonon–impurity scatterings), and the sum is over all the relevant phonon vectors (**q**) and modes, as well as electronic bands (*n*) and wavevectors (**k**). In standard metallic (accumulation) regime, *S*_g_ is only relevant in the narrow *T* interval (typically around *T~θ*_D_/5, *θ*_*D*_ is the Debye temperature) where *T* is large enough to activate a substantial number of phonons, but not so large as to make phonon–phonon scattering (*τ*_ph_^−1^*~T*^3^) dominant over EPC, as for *τ*_ph_^−1^>>*τ*_ep_^−1^ *S*_g_ quickly vanishes.

This scenario is dramatically altered in case of strong depletion. First, electron localization largely suppresses conductivity, thus favouring large *S*_g_, according to equation (1); at variance, *S*_d_ has a much weaker dependence on *σ*_*j*_ (see Supplementary equation (32) and the related discussion in the Supplementary Note 6), and cannot be amplified as much by a decrease of conductivity. Second, EPC may become very large in the so-called ‘dirty limit’, that is for electron diffusion lengths *L* much shorter than phonon wavelengths (*qL≪*1)^23^,^35^. In particular, this condition holds for narrow two-dimensional electron systems^41^ in the confined direction (for example, *z*), as strong EPC occurs for any *q*_*z*_*≪*1*/t* (*t* being the electron liquid thickness), thus any phonon wavevector contributes, eventually up to the Debye frequency. In other words, in the limit *t*→*0* EPC may be large since even very short-wavelength acoustic phonons can couple to electrons. A further burst to EPC may come from the polar character of the system, which can lead to a large coupling between electrons and the electric field associated with acoustic phonons of very-long wavelength. The peculiar characteristics of two-dimensional electron liquid (2DEL) in LAO/STO under large negative gate voltage are thus highly favourable to the occurrence of strong EPC.

**Figure 3:**
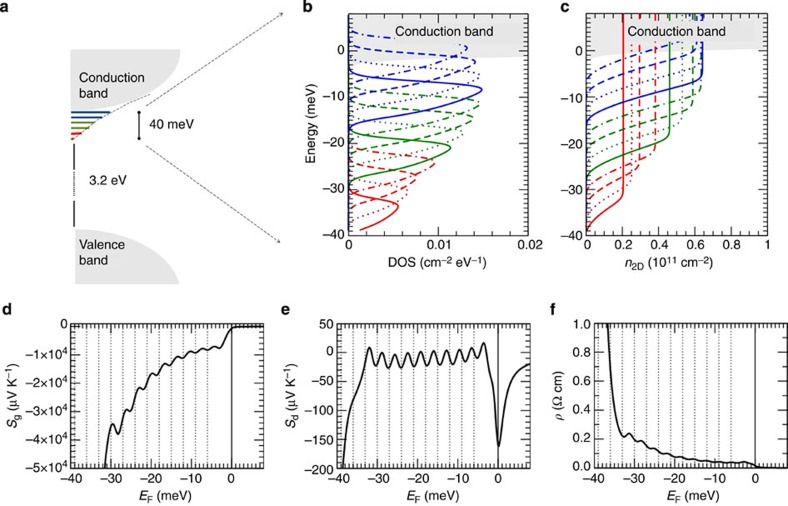
Electronic band structure of the two-dimensional electron liquid (2DEL) emerging from the experimental results, and calculated transport properties. (**a**) Sketch of the model band structure purposely built to reproduce the experimental results. Grey areas indicate valence and conduction states; the coloured lines below the conduction states represent a tail of localized states. (**b**) Actual density of states (DOS) of the model band structure considered for the calculations. The shaded grey area is the DOS relative to the conduction band bottom (CBB) of *t*_*2g*_ *d*_*xy*_ orbital character. Below the CBB lies a tail of 12 localized states, placed at regular intervals of 3 meV from each other, indicated by different colours and type of lines. From the bottom: red solid, dotted, dashed, dot-dashed and then the same sequence repeated in green and blue. Zero energy is fixed at the CBB. (**c**) Integrated DOS per unit area. The DOS is normalized to obtain for the total charge density hosted by the 12 localized states, *n*_2D_=6 × 10^11^ cm^−2^, that is, the Hall-measured charge depleted by field-effect in the interval *V*_g_=−14 V, −7 V, where the huge Seebeck oscillations are visible. (**d**) Phonon-drag calculated for the model DOS. The dotted vertical lines indicate the bottom energy of each localized state, the solid line is the CBB. *S*_g_ oscillates at each intersection of *E*_F_ with the bottom energies. (**e**) Diffusive Seebeck: like *S*_g_ it oscillates in correspondence with the depletion of each localized state, but in absolute value is about three orders of magnitude smaller than *S*_g_. (**f**) Electric resistivity *ρ* in 3D. To obtain the sheet resistance, *ρ* must be rescaled by the 2DEL thickness *t*, that is, *ρ*=*R*_sheet_·*t*.

As a quantitative assessment of our analysis, we calculate the Seebeck coefficient and resistivity for a multiband effective-mass model (details are given in the Supplementary Notes 4–8). The model includes a single delocalized conduction band of *t*_*2g*_ *d*_*xy*_ symmetry, which is sufficient to describe the low-density regime *n*_2D_ *<*10^13^ cm^−2^ according to first-principles calculations^4^. Below the conduction band bottom (CBB), we insert (see Fig. 3a–c) a series of low-density, low-mobility polaronic states (for brevity called ‘localized’ hereafter), with the intent of mimicking a disorder-induced Anderson tail, consistent with the scenario proposed in previous works^7^,^8^,^10^. Our model is also consistent with a scenario dominated by Mott localization, often invoked to explain other correlation phenomena observed in this system (see for example, capacitance enhancement described in ref. 13), as electrons can localize on Ti^3+^ sites at very low charge density, eventually helped by structural deformations (that is, polarons), even in the absence of disorder. As the measured Seebeck coefficient shows 12 oscillations in the range between −7 and −14 V, and the Hall measurement indicates in this voltage range a modulation of the carrier density of *n*_2D_~6 × 10^11^ cm^−2^, in the model we include 12 localized states, whose integrated DOS amounts to *n*_2D_~6 × 10^11^ cm^−2^. The basic characteristics of these states (energies, effective masses, bandwidth) are set to qualitatively reproduce the measured Seebeck in terms of oscillation frequency and amplitudes, as well as the Seebeck absolute value. The model is finalized to reproduce the following scenario: at zero voltage, the lowest conduction band is occupied by a mobile carrier density (*n*_2D_~1.2 × 10^13^ cm^−2^) similar to that usually found in LAO/STO. Then, it is assumed that the substantial effect of a negative *V*_g_ is the progressive charge depletion, thus the progressive lowering of *E*_F_, so that mapping *S(E*_F_) is equivalent to mapping *S(V*_g_) (additional details are given in the Supplementary Note 8).

In Fig. 3, the calculated *S(E*_F_) and *ρ(E*_F_) reproduce qualitatively the most important features observed at varying *V*_g_. In the experiment, negative *V*_g_ causes mild changes in *R*_sheet_ and *S* up to a certain threshold, which we can dub ‘regular behaviour’. In our interpretation, this threshold corresponds to the point where *E*_F_ reaches the CBB edge (zero energy in the Figure 3d–f). For *E*_F_≤*E*_CBB_, a dramatic upturn begins: *E*_F_ enters the region of localized states, *ρ* rises to Ωcm values and *S*_g_ (negligibly small in accumulation) quickly bursts up to 10^5^ μV K^−1^ in magnitude. During the *E*_F_ descent through the series of increasingly localized states, *S*_g_ oscillates in correspondence with the crossing (depletion) of each state. *S*_d_ also oscillates in correspondence with localized states, but with several fundamental differences from *S*_g_: (i) the magnitude of *S*_d_ (~10^2^ μV K^−1^) is in the usual range for LAO/STO (or even bulk SrTiO_3_) at low *T*, whereas *S*_g_ is 3–4 orders of magnitude larger; (ii) *S*_g_ increases rapidly in magnitude with the lowering of *E*_F_, as a consequence of the *1/*σ dependence, whereas the oscillation-averaged *S*_d_ changes very smoothly with *E*_F_; (iii) the amplitude of *S*_d_ oscillations is comparable with the averaged *S*_d_ value itself. Points (ii) and (iii) make *S*_d_ incompatible with the monotonic descent observed in the experiment. These aspects (discussed in detail in the Supplementary Note 8) are derived from the fundamental characteristics of *S*_d_, not from the specific approximation used in the model, thus they represent sound arguments to rule out diffusive Seebeck as the main contribution to the values measured in the strongly depleted regime. We remark that other forms of transport typical of localized carriers (variable-range hopping and thermal activated hopping) were considered as a possible source of the observed oscillations, but they were found incompatible with either the huge oscillating Seebeck or the resistivity values.

Notice that in the simulation *ρ* also shows oscillations, which are not observed experimentally. A non-oscillating behaviour of *R*_sheet_*(V*_g_) in the presence of oscillations in *S(V*_g_) has been previously noticed in 2DEGs (GaAs/AlGaAs^42^,^43^ and MoS_2_ (ref. 36)). We believe that this is a consequence of the different nature of the two measurements (open- versus closed-circuit for *S* and *R*, respectively) and the non-ohmic behaviour of resistivity in the strongly depleted regime^5^ (a more extended discussion can be found in the Supplementary Note 2).

In conclusion, we measure field-effect transport and thermoelectric properties of the 2DEL at the LAO/STO interface in a wide range of gate field values. Seebeck measurement is an exceptionally sensitive tool for the exploration of highly confined carriers in oxide heterostructures, as it displays a clear signal even for electronic states characterized by extremely low charge density and mobility, and capable to resolve electronic structures in the lower than meV range at very low temperature. In this respect, thermopower seems to be a far more effective probe than any photoemission techniques nowadays available (see Supplementary Note 3).

At low-*T*, for negative gate voltage larger in magnitude than a given threshold, the measured thermopower displays a diverging, oscillating behaviour with negative values that are unprecedented in the literature. With the help of model results, we interpret this finding as being due to the regime change from normal metal to localized electron behaviour, corresponding to the *E*_F_ descent below the conduction band, and across a series of lower lying localized electronic states, characterized by large resistivity, large EPC, and in turn huge measurable phonon drag.

This huge-Seebeck transient phase can be considered incipient of the insulating regime: it lives in a narrow charge density interval between the band-like metallic density (*n*_2D_≥10^13^ cm^−2^) and the insulating phase (*n*_2D_≤8–9 × 10^12^ cm^−2^), so that only a handful of these states (recognizable by the number of *S* oscillations) are visible. If the polarization catastrophe scenario is correct, we expect that a further lowering of *E*_F_ would bring us right into the insulating phase where a large quantity of localized charge (*n*_2D_~10^14^ cm^−2^) is present. We argue that this charge is associated to diverging *S* values and conductivity too low to be detected by any possible means. Thus, the thermopower oscillations measured in this work are a strong, direct signature of the presence of localized states in the LAO/STO system.

## Methods

### Sample preparation and measurement setup

The samples are prepared as described in detail in the ref. 44. Pulsed laser deposition is used to grow an epitaxial layer of LaAlO_3_ (4–10 unit cells thick) on a (001)-oriented TiO_2_-terminated SrTiO_3_ substrate. The substrate temperature is about 800 °C and the oxygen pressure 10^−4^ Torr. The laser fluence is estimated at 0.6 J cm^−2^. Reflection high-energy electron diffraction intensity oscillations and patterns show layer-by-layer growth and good crystalline quality. The growth rate is around 60 laser shots per unit cell. After deposition, the oxygen pressure is raised to 200 mbar and the sample is kept at 500 °C for 1 h before being cooled down to room temperature. X-ray diffraction patterns confirm that the LaAlO_3_ layers are epitaxial and crystalline while atomic force microscope images show atomically flat terraces on the LaAlO_3_ surface, with terrace-edge heights corresponding to one layer of LaAlO_3_. Other details about sample preparation are found in refs 45, 46, 47.

The samples measured in this work behave in a very similar way, so that data on only one of them are presented. One sample is patterned in the shape of a Hall bar (channel width 1.5 mm) for better definition of geometrical factors and is used as a reference to confirm the exact relationship between measured properties. The measurement configuration sketched in Fig. 1a shows that the a.c. heat flows along the 010 direction (the longer side) of the 10 × 5 mm^2^ crystal. The typical power fed to the ac heater is ~1 mW. The voltage contact separation, equal to thermocouple separation, is 1.5–3 mm. The back gate electrode is a 3 × 5 mm^2^ gold pad evaporated on the backside of the 0.5-mm-thick substrate. The leakage current to the gate electrode is always monitored to be below 10 nA during the *S* measurements.

The Seebeck coefficient is measured in a home-made cryostat, from 4.2 K to room temperature, using an ac technique^29^. The period of the sinusoidal power supplied to the sample is 150 s and the applied thermal gradient is around 0.3 K across a distance of ~2 mm. Hall effect and resistivity data are measured in a Quantum Design PPMS system, from 2 K to room temperature and in magnetic field up to 9 T. Both dc and ac techniques are used. In particular, in the depletion regime, the ac lock-in technique is preferable, in that it allows very low currents (~10 nA) to be applied and pinch-off problems to be avoided.

The electrical and thermoelectrical responses to field effect of all the samples exhibit the general features presented in this work, in particular the divergent and oscillating *S* in the depletion regime. Nevertheless, some specific details (threshold voltage for the occurrence of the depletion regime, as well as exact magnitude of *S*) may vary from sample to sample and from thermal cycle to thermal cycle. We believe that this behaviour originates from the fact that the electrostatic landscape around the 2D electron liquid may change significantly, because of empty and filled traps within the SrTiO_3_ substrate. This may affect the screening of the gate electric field and thus may be the primary source of changing threshold voltage and details of experimental curves. On the other hand, reproducible *S*(*V*_g_) curves are obtained after a forming protocol of *V*_g_ cycle at low temperature^33^, carried out by applying, at low temperature, first the highest positive voltage *V*_max_ (usually ~200 V) to be used and then sweeping the voltage between *V*_max_ and *V*_min_. This procedure allows reproducible measurements to be obtained, even sweeping *V*_g_ back and forth, as long as the sample is maintained at low temperature. The stability and reproducibility of Seebeck measurements after the forming protocol is also checked by measuring Seebeck signal for hours at fixed temperature and gate voltage, which yields constant curves.

In any case, considering all the different sets of measurements, it is clear that all the measurements present the same striking common features, that is evenly spaced oscillations as a function of gate voltage and diverging Seebeck coefficient in the tens of mV K^−1^ range in the depletion regime, which support the general character of our results and their interpretation.

We point out that there is a clear discrepancy between, on the one hand, the low T limit values in electrical and thermo-electrical transport curves as shown in Fig. 2a and, on the other hand, the zero gate voltage values of thermo-electrical and electrical transport properties as shown in Figs 1b and 2b, respectively. Indeed, the *S* value at the lowest temperature in Fig. 2a is around *−*240 μV K^−1^ slightly different from the *V*_g_*=0* value of Fig. 1b. Similarly, the *n*_2D_(*V*_g_=0) value in Fig. 2b is much smaller than the low temperature value displayed in the third panel of Fig. 2a. This is due to the fact that the electron state at the interface setup by the forming protocol is stable only at the lowest temperatures. Data in Fig. 1b and Fig. 2b are measured after the forming protocol, whereas in Fig. 2a, data are measured before the forming protocol.

### Theoretical modelling

Diffusive thermopower and electric resistivity are calculated through the Boltzmann equation in the relaxation time approximation^48^. The electronic structure is simulated using a variant of the multi-band effective mass modelling previously employed to describe STO/LAO^30^, and the electronic relaxation time is modelled in terms of common energy-dependent analytic formulae (the Brooks-Herring formula for impurity scattering and the deformation potential approach for acoustic phonon scattering). Phonon-drag formulation is based on the coupled Boltzmann equations for electrons and phonons, in relaxation time approximation^40^,^41^. As the low-temperature limit is required, only the electron-acoustic phonon scattering is included in the phonon drag. Acoustic phonons are modelled according to the deformation potential approach, plus a piezoelectric scattering to account for the polar character of STO. The theoretical model is built to reproduce n-doped STO bulk, even with the presence of localized states below the conduction band (see Supplementary Figs 2 and 3). LAO/STO quantum well DOS is modelled with 12 equally spaced localized levels lying below the CBB. In Supplementary Fig. 4, the individual contributions of each electronic state to phonon drag, diffusive Seebeck and conductivity are shown, whereas main features of each level in terms of effective mass and electronic bandwidth are reported in Supplementary Table 1. Finally, the electron-phonon scattering rate at different doping level is reported in Supplementary Fig. 5. The formulation is displayed in full detail in the Supplementary Note 4.

## Author contributions

Samples were deposited by D.L. and A.F. Transport and thermoelectric measurements were performed by I.P., F.T. and D.M. Theoretical calculations and modelling were carried out by A.F. P.D. and V.F. All the authors contributed to data interpretation and to the manuscript preparation and approved the final version of the manuscript.

## Additional information

**How to cite this article:** Pallecchi, I. *et al*. Giant oscillating thermopower at oxide interfaces. *Nat. Commun.* 6:6678 doi: 10.1038/ncomms7678 (2015).

## Supplementary Material

Supplementary Information Supplementary Figures 1-5, Supplementary Table 1, Supplementary Notes 1-8 and Supplementary References
