# Supplementary material for: Giant oscillating thermopower at oxide interfaces
Source: Nat Commun. 2015 Mar 27;6:6678. doi: 10.1038/ncomms7678 (PMC4389223; doi:10.1038/ncomms7678)
Supplement: Supplementary Information — Supplementary Figures 1-5, Supplementary Table 1, Supplementary Notes 1-8 and Supplementary References [file ncomms7678-s1.pdf]

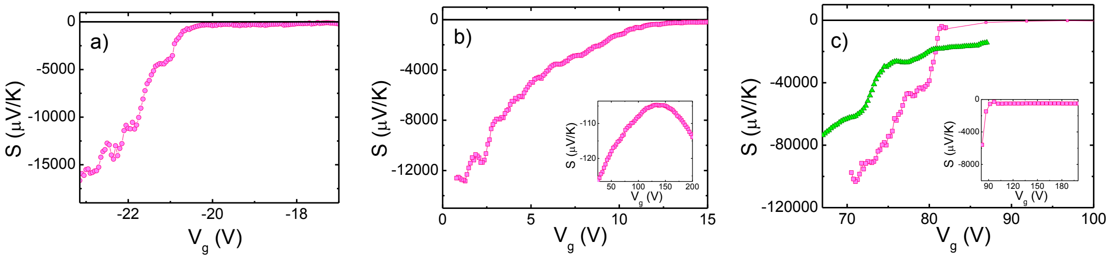

Supplementary Figure 1: Seebeck coefficient versus gate voltage measured at 4.2K in other LAO/STO interfaces, whose parameters are listed in the text.

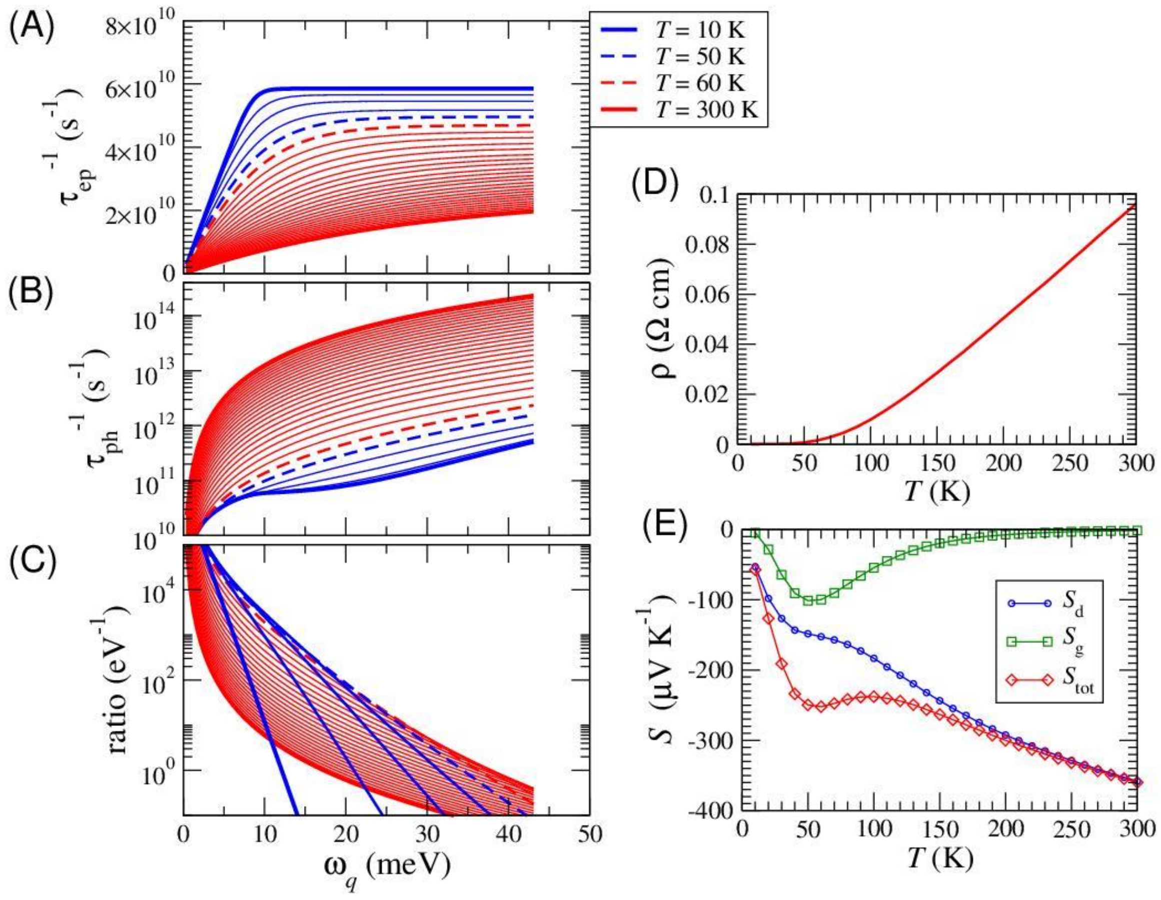

Supplementary Figure 2: Calculations for n-doped STO bulk (n_{3D}= 2×10^{19} cm^{-3}) represented by three t_{2g} conduction bands (A): electron-phonon scattering frequency as a function of phonon energy calculated from Equation (30); different curves span a temperature range from 10 K to 300 K in steps of 10 K. Blue to red color change highlights the regime change from growing to decreasing phonon-drag. (B): total phonon scattering frequency calculated adding the electron-phonon to the other contributions given in Equation (31). (C): relative scattering ratio (Equation (38)): it grows with T up to 50-60 K (highlighted by the dashed lines), and then it starts to fall and decreases smoothly up to vanishing at room T. (D): DC resistivity, signaling metallic behavior. (E): total Seebeck and decomposition in diffusive and phonon-drag contributions.

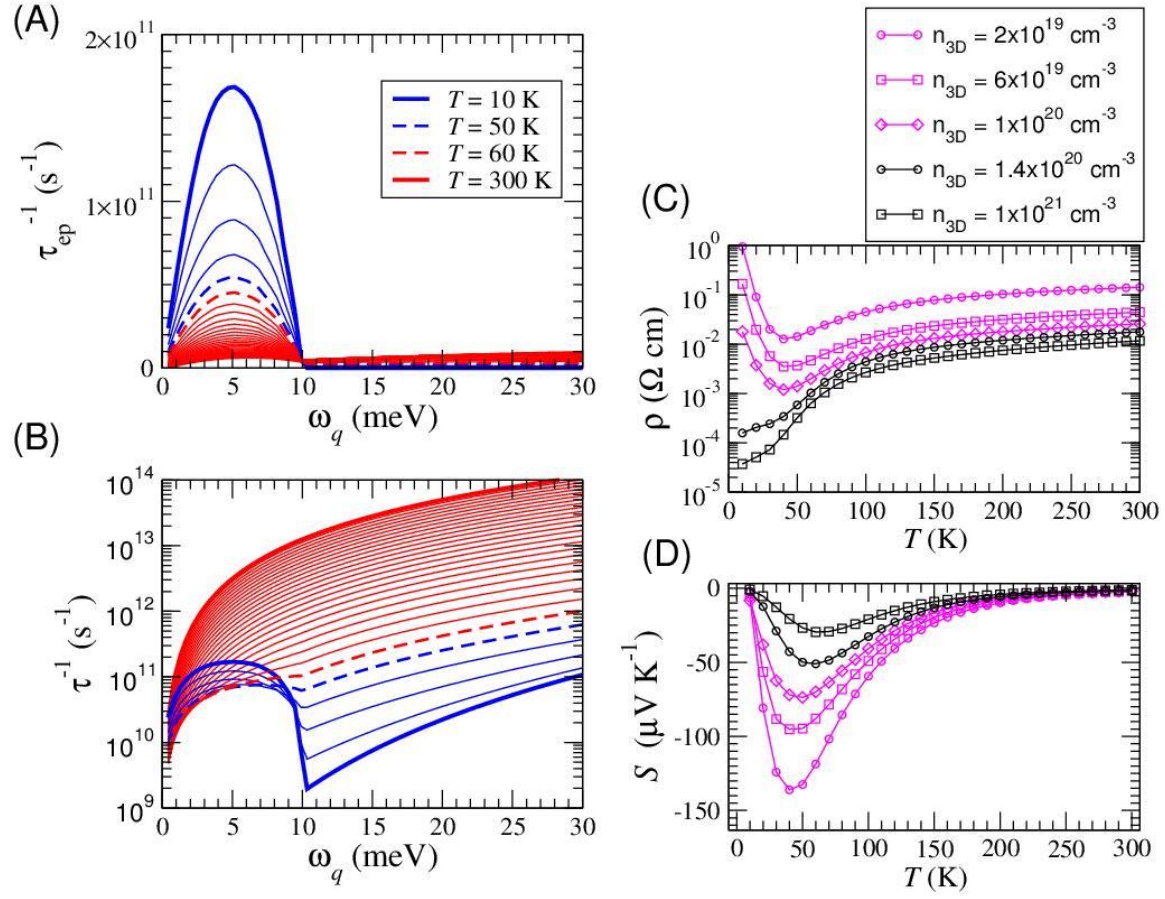

Supplementary Figure 3: Calculations for n-doped STO bulk whose electronic structure consists in three t_{2g} conduction bands and one localized state lying 10 meV below the CBB (see text) (A): electron-phonon scattering frequency as a function of phonon energy calculated from Equation (30) at n_{3D}= 2×10^{19} cm^{-3}; different curves span a temperature range from 10 K to 300 K in steps of 10 K. (B): total phonon scattering frequency at n_{3D}= 2×10^{19} cm^{-3} calculated as the sum of electron-phonon scattering in Equation (28) and other phonon scatterings given in Equation (29). (C): resistivity calculated at different doping concentrations (E): phonon-drag calculated at different doping concentrations

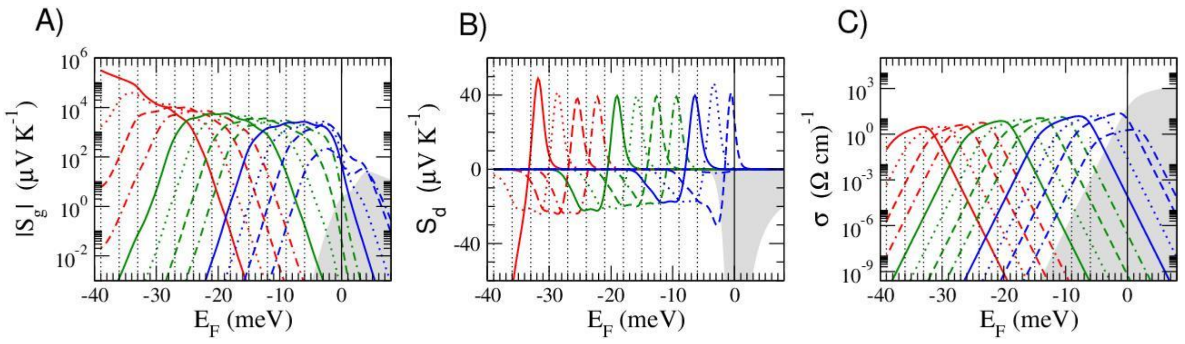

Supplementary Figure 4: Individual contributions of each electronic state to phonon-drag, diffusive Seebeck, and conductivity, calculated for the model DOS in Figure 3 A): Phonon-drag amplitudes due to individual electronic states. The dotted vertical lines indicate the bottom energy of each localized state, the solid line is the CBB. Line colors and styles relate each contribution to the corresponding state DOS in 3B). The gray-shaded area is the contribution of the lowest conduction band (gray-shaded area). **B):** Contributions to S_d from each state. In contrast with S_g, the contributions due to the localized states (except for the conduction band) always cross the zero. C): State-by-state contributions to 3D conductivity; $\sigma_{sheet} = \sigma \cdot t$.

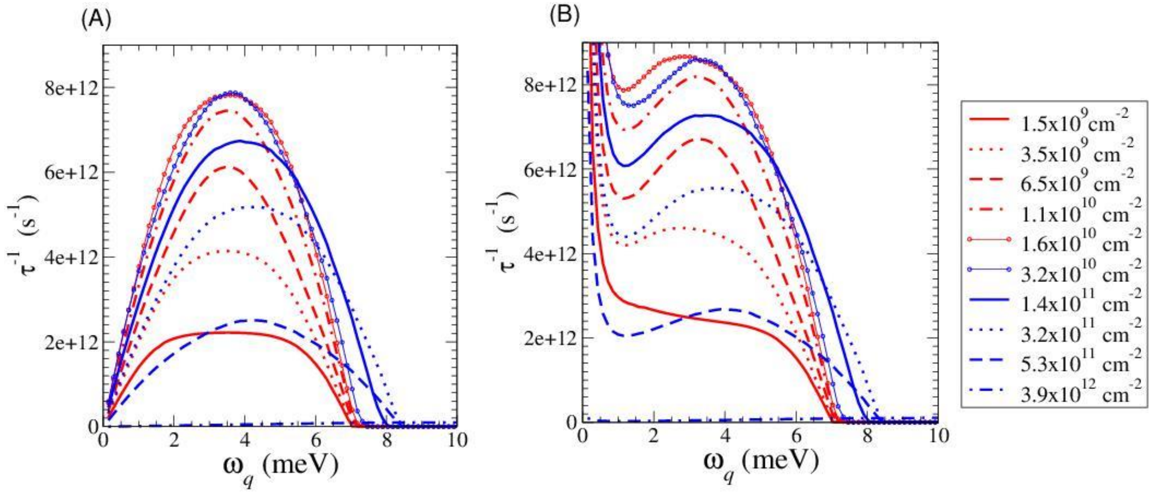

Supplementary Figure 5: Electron-phonon scattering rate calculated from Equation (30) at fixed *T*=4.2 K and varying doping concentration mimicking the progressive charge depletion for increasingly negative gate voltage. Different curves are for different Fermi energies (i.e. doping), spanning the range of localized states illustrated in Figure 3 of the main article. The red solid curve is for the lowest E_F; starting from low doping, the scattering first increases with E_F (red curves) and then it decrease (blue curves). (A): only the deformation-potential contribution is included in the electron-phonon scattering (first term of Equation (22)). (B) both deformation potential plus piezoelectric scattering are included, according to Equation (22).

SUPPLEMENTARY TABLES

Supplementary Table 1: Bottom energy (ε_b), effective masses, and bandwidth (W) of the energy levels used to model the n-doped LAO/STO interface under large negative gate voltage. The conduction band bottom (CBB) is placed at zero. Below CBB, a series of 12 localized states located at 3 meV from each other span a range of 40 meV. Effective masses increase and bandwidths decrease while going from higher to lower energies.

ε_b (meV)	m_i^* (m_e)	W (meV)
0	0.7, 0.7, 8.0	1000
-6	6, 6, 20	8.4
-9	7, 7, 20	8.4
-12	8, 8, 20	8.4
-15	9, 9, 20	8.4
-18	10, 10, 20	8.4
-21	11, 11, 20	8.2
-24	12, 12, 20	8.0
-27	13, 13, 20	7.8
-30	14, 14, 20	7.6
-33	15, 15, 20	7.4
-36	16, 16, 20	7.2
-39	17, 17, 20	7.0

Supplementary Note 1: Remark on the reproducibility of diverging and oscillating behavior of S in the depletion regime in different LAO/STO samples.

Here below we show other $S(V_g)$ measurements carried out on different LAO/STO interfaces. The main features discussed in this work – i.e. diverging and oscillating behavior of S in the depletion regime and change of slope in electrical and thermo-electrical transport curves at the crossover between accumulation and depletion regimes - are present in all cases. In panel a) of Supplementary Figure 1, the measurement is carried out on a sample having similar sheet carrier density and sheet resistance as that presented in the main text (namely the regime of S oscillations corresponds to $|S|\approx15$ mV/K, $R_{sheet}\approx10$ KΩ and $n_{2D}\approx6\times10^{12}$ cm^{-2}, while in the accumulation regime $|S|\approx200$ μV/K, $R_{sheet}\approx530$ Ω and $n_{2D}\approx2.7\times10^{13}$ cm^{-2}). In panel b), it is presented the behavior of a LAO/STO interface, where the regime of S oscillations corresponds to $|S|\approx13$ mV/K and $R_{sheet}\approx100$ KΩ, while in the accumulation regime $|S|\approx100$ μV/K, $R_{sheet}\approx1$ KΩ and $n_{2D}\approx3\times10^{13}$ cm^{-2}. Finally, in panel c), it is presented the behavior of a different LAO/STO interface, where the regime of S oscillations corresponds to $|S|\approx50$-100 mV/K and $R_{sheet}\approx100$ KΩ - 10 MΩ, while in the accumulation regime $|S|\approx500$ μV/K, $R_{sheet}\approx10$ KΩ and $n_{2D}\approx2\times10^{13}$ cm^{-2}. We note that the crossover between accumulation and depletion regimes may occur at very different threshold values of the gate voltage in different samples after the poling protocol, depending on the electrostatic landscape between the 2DEG and the back gate electrode, namely it is around zero voltage for the sample presented in the main text, around V_g=-20V for the sample in panel a), around V_g=+10V for the sample in panel b) and around V_g=+80V for the sample in panel c). For the latter sample, the threshold gate voltage even shifts among different poling runs.

Supplementary Note 2: Remark on absence of oscillations related to localized levels in the $R_{sheet}(V_g)$ curves.

The simulated resistance shows oscillations in correspondence of the Fermi level crossing the localized levels, as does the Seebeck curve. However, these oscillations are not observed experimentally. We first point out that, from the experimental point of view, we carried out the resistance measurements with the highest possible level of accuracy and reliability, trying different configurations. In particular, we measured several samples in the maximum possible range of V_g, compatible with input impedance of nanovoltmeters, R_{sheet} measurements were carried out using both d.c. (either applying alternating polarity or fixed polarity) and a.c. (lock in) techniques, we measured both voltage drops with a current bias and flowing current with a voltage bias, we took care of avoiding pinch off condition, we changed measuring parameters in such a way that the voltage drop in the transport measurement was similar to or even smaller than the voltage drop in the thermopower measurement, we measured data points closely spaced in the V_g axis. In all our attempts, oscillatory behavior was never observed in the resistance curves of any of the investigated samples. We rule out the slight intrinsic spatial inhomogeneity in these systems as a cause for the absence of oscillations in R_{sheet} curves, as it should have a smearing effect on S and R_{sheet} curves alike. On the other hand, similar discrepancy between $R_{sheet}(V_g)$ and $S(V_g)$ curves is not noticed here for the first time. In other systems, e.g. GaAs/AlGaAs [1,2] and MoS_2 [3], oscillations and divergence of S at negative V_g were observed, without corresponding oscillations in R. The key difference between Seebeck and resistance lies in the respective nature of these two measurements, namely the former is an open-circuit measurement with no net flow of electric charge, where the effect of the applied thermal gradient is counterbalanced by a voltage drop, while the latter is a closed-circuit measurement with injected current. We suggest that these differences, combined with strong non ohmic behavior of LAO/STO in the depletion regime, widely observed in literature [4], play a decisive role in washing out features associated to localized level filling in the resistance measurements. Similar non linear effects may be also responsible for the

absence of oscillations in several resistivity measurements reported in literature [1,3], where diverging *R* and *S* in the depletion regime are likely associated to non ohmic transport mechanisms. On the contrary, literature works reporting oscillations of *S* versus V_{g}, associated to very low and non-diverging resistance versus V_{g} curves, evidence oscillations both in Seebeck and resistance curves [5,6].

Oppositely, the measurement of *S* is unobtrusive and permits an ultra-refined scan of the unperturbed, closely-spaced polaronic densities lying below the band edge. Hence, the oscillations of *S* should be thus ascribed to a paramount advantage of thermoelectric spectroscopy: the capability to probe extremely small changes in the charge density, without introducing significant perturbations. For this aspect, thermopower is far superior not only to resistivity measurements, but also to most of nowadays available spectroscopic techniques.

Supplementary Note 3: On the sensitivity of Seebeck measurement with respect to other spectroscopies

In the main article we claim that our Seebeck measurement is the first direct evidence of 'nearly-localized' states lying below the conduction band bottom. We need to specify the exact meaning of this strong statement, distinguishing what for us is 'direct' and 'indirect'.

Indirect evidences of charge localization under negative gate voltage are in fact abundant in literature, ranging from capacitance enhancement and negative compressibility [7] to metal-insulator transition at a specific charge threshold, magnetic correlations [8], and phase separation [9]. Furthermore, there is a number of spectroscopic measurements (see e.g. HAXPES spectra in [10]) where a peak of Ti^{3+} character is clearly detected, attributed to localized electrons occupying a small fraction of Ti sites. All these evidences consistently suggest the presence of localization in some form in the highly depleted regime (typically below few 10^{12} cm^{-2}). On the other hand, what we define a 'direct' evidence has to do with a detailed scanning of the electronic structure of these states. This aspect has been, to our knowledge, lacking so far, and in fact, we claim that the Seebeck oscillations measured in this work are the first direct evidence of this sort. There are two major reasons at the basis of this unprecedented achievement:

a) the specific characteristics of the thermoelectric measurement, sensitive to charge density fluctuations as small as 10^{10} cm^{-2} which translates, in terms of energies, to a resolution of the order of meV and below. This is a resolution that few spectroscopies can achieve, in practice.

b) the induction of a strongly-depleted regime: as long as the metallic regime is retained, no technique could be capable to distinguish the presence of tiny densities of polaronic levels. The *complete* depletion of the conduction band is 'conditio-sine-qua-non' for the emergence of localized behavior in transport, thermoelectric, or even electric and optical measurements.

Consider for example ARPES measurements, which is the prototypical approach to explore the electronic structure of a system: experiments carried out so far on LAO/STO have employed soft-X ray

ARPES which at its best is performed with a resolution of 50 meV [11,12,13], i.e. a resolution way too low to distinguish levels separated a few meV one from each other and from the band bottom, as those described in our work. Furthermore, the well known "waterfall effects" creating blurred trails below the band bottom would make any attempt to distinguish features from these localized states totally hopeless. Finally, we point out that UV-ARPES with less than 10 meV resolution could be done, but the electron escape depth would be as short as few Angstrom, thus not enough to overcome the LAO barrier. More generally for what concern photoemission measurements, a long list of works can be found for LAO/STO with different techniques (HAXPES, RIXS, XPS, XAS, etc.) but after a careful exploration of this massive literature, we can safely conclude that none of those present the suited characteristics in terms of energy and charge density resolution to reveal the electronic structure at the same level of detail as that inferred from our Seebeck measurements.

A possible suitable technique to detect such localized states, could be scanning tunneling spectroscopy. With this technique, operating at low temperature (5K) it could be possible in principle to resolve electronic levels that are even more closely spaced than the ones observed in our experiment. However the design of such an experiment should face several difficulties, mostly related to the buried interface and to the contributions to the DOS coming also from surface and defect states. To the best of our knowledge, only one paper in literature shows scanning tunnel spectroscopy on LAO/STO but, in that case, data were averaged on 75 meV [14], so that features on the meV scale were not visible.

Supplementary Note 4: Phonon-drag model

Introduction - Starting from the general expression for phonon-drag [15,16,17,18,19,20], we develop a specific formulation adapted to our means. We use a formalism based on 3D electronic structure coupled with 3D phonons, instead of the more common 2D electronic structure coupled with 3D phonons. Use of 3D formulas for the electrons is motivated by the need to treat on equal footing the bands and localized states, without a-priori assumption on the dimensionality. Of course, the electronic structure is fully anisotropic, and the 2D limit is easily recovered by imposing large effective masses in the interface-orthogonal direction. The multi-band effective mass modeling includes both delocalized conduction states and localized polaronic states (the latter having renormalized mass [21] and finite bandwidth) as shown in Figure 3 of the main text.

Since we are only interested in the low-T limit, only the interaction of electrons with acoustic phonons will be considered as a source of phonon-drag. The interaction is treated according to the deformation potential approach plus piezoelectric scattering to account for the polar character of the system. For simplicity, only one acoustic phonon branch and intra-band electron-phonon scattering is considered. These approximations may be seriously detrimental for detailed quantitative predictions, but are sufficient for the purpose of reproducing the order of magnitude and major features of the observed behavior.

From the coupled Boltzmann equations for electrons and phonons, the phonon-drag in the j direction is expressed as:

$$S_j^{\mathrm{g}} = \left(\frac{2e}{\sigma_j V K_{\mathrm{B}} T^2} \right) \sum_{n\mathbf{k},n\mathbf{k}',\mathbf{q}} \hbar\omega_{\mathbf{q}} \left(\frac{\Gamma_{n\mathbf{k},n\mathbf{k}'}(\mathbf{q})}{\tau_{\mathrm{ph}}^{-1}(\mathbf{q}) + \tau_{\mathrm{ep}}^{-1}(\mathbf{q})} \right) V_j(n\mathbf{k},n\mathbf{k}',\mathbf{q}) \qquad (1)$$

where the factor 2 accounts for spin degeneracy, e is the electron charge, σ_j the electron conductivity, and V the volume; $\mathbf{q}$ and $\omega_{\mathbf{q}}$ are phonon wavevector and frequency, respectively; $\Gamma_{n\mathbf{k},n\mathbf{k}'}(\mathbf{q})$ is the electron-phonon scattering rate (EPR) in k-space, $\tau_{\mathrm{ep}}(\mathbf{q})$ the electron-phonon relaxation time, whereas $\tau_{\mathrm{ph}}(\mathbf{q})$ includes all the other relevant phonon scattering processes (phonon-phonon scattering, boundary scattering, impurity scattering, etc.). V_j is a velocity factor:

$$V_j(n\mathbf{k},n\mathbf{k}',\mathbf{q}) = v_j(\mathbf{q})\left[\tau(\mathbf{k})v_j(n\mathbf{k}) - \tau(\mathbf{k}')v_j(n\mathbf{k}')\right] \quad (2)$$

where $\tau(\mathbf{k})$ is the electronic relaxation time, $v_j(\mathbf{q})$ and $v_j(n\mathbf{k})$ phonon and electron velocities, respectively. From Equation (1) it is apparent that phonon-drag essentially depends on two dominant factors: the inverse electric conductivity and the ratio of electron-phonon to total phonon scattering. Clearly, phonon-drag only matters if EPR is non-discardable with respect to the other phonon scattering processes. Following Cantrell notations [16], the inverse electron-phonon relaxation time (i.e. electron-phonon scattering frequency) is:

$$\tau_{\mathrm{ep}}^{-1}(\mathbf{q}) = -\left(\frac{2}{N_{\mathbf{q}}^{'}K_{\mathrm{B}}T}\right)\sum_{n\mathbf{k},n\mathbf{k}'}\Gamma_{n\mathbf{k},n\mathbf{k}'}(\mathbf{q}) \quad (3)$$

$$\Gamma_{n\mathbf{k},n\mathbf{k}'}(\mathbf{q}) = f_{n\mathbf{k}}\left(1 - f_{n\mathbf{k}'}\right)N_{\mathbf{q}}A(\mathbf{q})\,\delta\left(\varepsilon_{n\mathbf{k}'} - \varepsilon_{n\mathbf{k}} - \hbar\omega_{\mathbf{q}}\right)\delta_{\mathbf{k}',\mathbf{k}+\mathbf{q}} \quad (4)$$

where $f_{n\mathbf{k}}$ and $N_{\mathbf{q}}$ are Fermi-Dirac and Phonon equilibrium distributions, respectively, $A(\mathbf{q})$ the electron-acoustic phonon coupling amplitude, and the two delta functions impose energy and momentum conservation for the absorption process (emission is accounted by the velocity factor). Equation (1) implicitly assumes the relaxation time approximation for phonons: the decrease of phonon population $N_{\mathbf{q}}^{Ph}$ due to phonon scattering is:

$$\frac{\partial N_{\mathbf{q}}^{Ph}}{\partial t} \approx -\left(\frac{\partial N_{\mathbf{q}}}{\partial q}\right)\left(\frac{1}{\tau_{\mathrm{ph}}(\mathbf{q})}+\frac{1}{\tau_{\mathrm{ep}}(\mathbf{q})}\right) \tag{5}$$

Velocity factor - In our model:

$$v_j(\mathbf{q}) = v_s \hat{q}_j; \qquad v_j(n\mathbf{k}) = \frac{\hbar k_j}{m_{nj}^*} \tag{6}$$

where v_s is the sound velocity, and m_{nj}^* the nth-band effective mass along j. From the linear-scaling approximation for the acoustic phonons we have $\omega_{\mathbf{q}} = \omega_q = v_s q$. Furthermore, we consider an isotropic phonon distribution, and put q_j^2=1/3 q^2. Solving for $\delta_{k+q,k'}$ and assuming for simplicity $\tau(\mathbf{k}) = \tau(\mathbf{k}')$ we thus have:

$$V_j(n\mathbf{k}, n\mathbf{k}+\mathbf{q}, \mathbf{q}) = -\frac{v_s q_j}{q}\frac{\hbar q_j}{m_{nj}^*}\tau(\mathbf{k}) = -\frac{\hbar v_s q}{3m_{nj}^*}\tau(\mathbf{k}) \tag{7}$$

The minus sign derives from the fact that for positive band curvature (thus electrons) the band velocity increases with **k**.

Energy-conserving delta function - To handle non isotropic effective masses, it is useful to introduce the following change of variables:

$$k_j = K_j\sqrt{\frac{m_{nj}^*}{m}}; \qquad q_j = Q_j\sqrt{\frac{m_{nj}^*}{m}} \tag{8}$$

where *m* is an auxiliary mass. Thus, band energies can be rewritten at 2nd order (setting the band bottom $\varepsilon_{n\mathbf{k}}^0$=0 for brevity):

$$\varepsilon_{n\mathbf{k}} = \frac{\hbar^2}{2}\left(\frac{k_x^2}{m_{nx}^*} + \frac{k_y^2}{m_{ny}^*} + \frac{k_z^2}{m_{nz}^*}\right) = \frac{\hbar^2 K^2}{2m} = \varepsilon_{nK} \quad (9)$$

$$\varepsilon_{n\mathbf{k}+\mathbf{q}} = \frac{\hbar^2(\mathbf{K}+\mathbf{Q})^2}{2m} = \frac{\hbar^2 K^2}{2m} + \frac{\hbar^2 Q^2}{2m} + \frac{\hbar^2 KQ\cos\theta}{m} = \varepsilon_{nK+Q} \quad (10)$$

where θ is the angle formed by vectors $\boldsymbol{K}$ and $\boldsymbol{Q}$. We then operate another change of variable from θ to X defined as:

$$X = \frac{\hbar^2 KQ\cos\vartheta}{m} \quad (11)$$

thus

$$\delta\left(\varepsilon_{n\mathbf{k}+\mathbf{q}} - \varepsilon_{n\mathbf{k}} - \hbar\omega_q\right) = \delta\left(X - X_0\right) \qquad X_0 = \hbar\omega_q - \frac{\hbar^2 Q^2}{2m} \quad (12)$$

The integral over X is only non vanishing for X_0 included in the integral limits $X_{min} = -\hbar^2 KQ/m$ and $X_{max} = \hbar^2 KQ/m$, that is:

$$-\frac{\hbar^2 KQ}{m} \leq \hbar\omega_q - \frac{\hbar^2 Q^2}{2m} \leq \frac{\hbar^2 KQ}{m} \quad (13)$$

the inequality can be rewritten:

$$\frac{\hbar^2(K-Q)^2}{2m} \leq \varepsilon_{nk} + \hbar\omega_q \leq \frac{\hbar^2(K+Q)^2}{2m} \quad (14)$$

which has a simple interpretation: after absorption the carrier energy must be higher (lower) than the band energy corresponding to antiparallel (parallel) $\boldsymbol{K}$ and $\boldsymbol{Q}$ orientation. For conduction (valence) electrons, only the right (left) inequality matters.

Sum over crystal momentum - The sum over **k** is transformed first into a 3D-integral, then changed to an integral over $\boldsymbol{K}$ in polar coordinates. We integrate over the equatorial angle of $\boldsymbol{K}$ space, and take the

azimuth angle as the θ between $\boldsymbol{K}$ and $\boldsymbol{Q}$, and then change θ to X. Solving for the energy delta function we finally obtain:

$$S_j^g = -\left(\frac{2\mathrm{e}\, v_s^2}{3(2\pi)^2 \sigma_\mathrm{j} \mathrm{k_B} \mathrm{T}^2}\right) \sum_{n=1}^{N_b} \frac{1}{m_{nj}^*} \sqrt{\frac{m_{nx}^* m_{ny}^* m_{nz}^*}{m}} \int dK\, K\, f_{\mathrm{n}K}\, \tau(\mathbf{k})$$

$$\times \sum_{\mathbf{q}} \frac{1}{Q} \frac{q^2 N_q A(\mathbf{q})}{\tau_\mathrm{ph}^{-1}(\mathbf{q}) + \tau_\mathrm{ep}^{-1}(\mathbf{q})} \left(1 - f_{\mathrm{n}K+\mathrm{Q}}\right) \qquad (15)$$

Here N_b is the number of bands. The minus sing comes from the velocity factor. Finally, K can be changed with the energy (Equation (10)):

$$S_j^g = -\left(\frac{2\mathrm{e}\, v_s^2}{3(2\pi)^2 \hbar^2 \sigma_\mathrm{j} \mathrm{k_B} \mathrm{T}^2}\right) \sum_{n=1}^{N_b} \frac{m}{m_{nj}^*} \sqrt{\frac{m_{nx}^* m_{ny}^* m_{nz}^*}{m}} \int_{\varepsilon_n^0}^{\varepsilon_n^0 + W} d\varepsilon\, f_\varepsilon\, \tau(\varepsilon)$$

$$\times \sum_{\mathbf{q}} \frac{1}{Q} \frac{q^2 N_q A(\mathbf{q})}{\tau_\mathrm{ph}^{-1}(\mathbf{q}) + \tau_\mathrm{ep}^{-1}(\mathbf{q})} \left(1 - f_{\varepsilon + \hbar\omega_q}\right) \qquad (16)$$

where ε_{nk}^0 and W_n are band bottom and bandwidth, respectively, and we have assumed, as customary, that the electronic relaxation time only depends on the wavevector through the energy.

Integration over phonon wavevector - The modulus of the rescaled variable $\boldsymbol{Q}$ in Equation (16) includes the band masses according to Equation (8); this would require to solve a 3D integration over the phonon wavevector. To simplify the calculation, we assume the following approximation:

$$Q \approx \sqrt{\frac{m}{\tilde{m}_n}}\, q; \qquad \tilde{m}_n \approx \frac{m_{nx}^* m_{ny}^* m_{nz}^*}{m_{nx}^* m_{ny}^* + m_{nx}^* m_{nz}^* + m_{ny}^* m_{nz}^*} \qquad (17)$$

where $\tilde{m}_n$ is the geometrically averaged effective mass. As showed later on, scattering amplitude A and phonon relaxation times τ_ph and τ_ep actually depend only on the modulus q; thus, we can easily transform the sum over $\mathbf{q}$ in Equation (16) in the integral, and solve it in polar coordinates:

$$S_j^g = -\left(\frac{V e\, v_s^2}{12\pi^4\hbar^2\sigma_j k_B T^2}\right)\sum_{n=1}^{N_b}\tilde{m}_{nj}\int_{\varepsilon_n^0}^{\varepsilon_n^0+W} d\varepsilon\, \tau(\varepsilon)\, f_\varepsilon$$

$$\times\int_0^{q_D} dq\left(\frac{q^3 N_q A(q)}{\tau_{\mathrm{ph}}^{-1}(q)+\tau_{\mathrm{ep}}^{-1}(q)}\right)\left(1-f_{\varepsilon+\hbar\omega_q}\right) \qquad (18)$$

Here the phonon wavevector is integrated up to the Debye frequency $q_D = k_B T_D / v_s\hbar$, where T_D is the Debye temperature (~500 K for STO [22]), and:

$$\tilde{m}_{nj} = \frac{1}{m_{nj}^*}\frac{m_{nx}^* m_{ny}^* m_{nz}^*}{\sqrt{m_{nx}^* m_{ny}^* + m_{nx}^* m_{nz}^* + m_{ny}^* m_{nz}^*}} \qquad (19)$$

Electron-phonon coupling - In the simplest treatment, the electron-acoustic phonon scattering for an isotropic phonon distribution can be taken as the sum of two terms [23,24]:

$$A(q) = \left(\frac{\pi D^2}{V\rho v_s}\right)q + \left(\frac{\pi e^2 v_s K_{\mathrm{em}}^2}{V\kappa_0\kappa}\right)\frac{q^3}{\left(q^2+q_0^2\right)^2} \qquad (20)$$

where D is the deformation potential, ρ the mass density, κ_0 and κ vacuum permittivity and dielectric constant, K_{em} the 3D-averaged electromechanical coupling, and q_0 the Debye screening length. The first term ("deformation potential scattering") describes the coupling of electrons with long-wavelength longitudinal acoustic waves treated as an homogeneous strain. The second ("piezoelectric scattering") is the additional contribution due to the coupling with the electric field produced by the strain. For a non-polar system (K_{em} =0) or in the limit of large doping concentration (i.e. strong Debye screening) the second term vanishes and only the deformation potential contributes to the acoustic scattering. In case of small screening (q_0 =0) and highly ionic compounds, on the other hand, the piezoelectric contribution (~1/q) may become dominant at small q. For some parameters appearing in Equation (22) the appropriate values to be used for our LAO/STO model are not easily determined, since these measurements may crucially depend on structural and chemical composition of samples, as

well as doping type and concentration, thus they should be considered as merely indicative. We take $K_{\mathrm{em}} = 0.37$, i.e. the average of planar and transversal components measured for a solid solution of Ti-based ceramics [25]. The dielectric constant under large negative gate is also difficult to be quantified reliably. It has been demonstrated [26] that below T=50 K a large negative gate field induces a polar transition in STO which sweeps away the quantum paraelectric state and largely reduces the screening capability. Tentatively, we use $k = 300$, that is the room-T value for STO bulk. Furthermore, we can assume a very long Debye length so that carrier screening is discardable ($q_0 \approx 0$). This is indeed a basic hypothesis for having strong electron-phonon scattering [27], i.e. in the low-density charge-localized limit the carrier mobility is so small that the piezoelectric interaction becomes unscreened. Finally for LAO/STO we use D=8.2 eV (used for GaAs in Ref. [16]) and $v_{\mathrm{s}} = 0.05\,\mathrm{m\,sec^{-1}}$(that is the 3D-average sound speed at low temperature [28]).Equation (22) is then inserted into Equation (20) to obtain:

$$S_j^g = -\left(\frac{\mathrm{e}\, v_s^2}{12\pi^3\hbar^2\sigma_{\mathrm{j}}\mathrm{k_B}\mathrm{T}^2}\right)\sum_{n=1}^{N_b}\tilde{m}_{nj}\int_{\varepsilon_n^0}^{\varepsilon_n^0+W} d\varepsilon\, \tau(\varepsilon)\, f_\varepsilon$$

$$\times \int_0^{q_D} dq \left(\frac{N_q}{\tau_{\mathrm{ph}}^{-1}(q)+\tau_{\mathrm{ep}}^{-1}(q)}\right)\left(C_{DP}\, q^4 + C_{PZ}\frac{q^6}{\left(q^2+q_0^2\right)^2}\right)\left(1 - f_{\varepsilon+\hbar\omega_q}\right) \qquad (21)$$

$$C_{DP} = \frac{D^2}{\rho v_s}; \qquad C_{PZ} = \frac{e^2 v_s K_{\mathrm{em}}^2}{\kappa_0 \kappa} \qquad (22)$$

which is finally integrated numerically.

Supplementary note 5: Phonon relaxation time

For the electron-acoustic phonon scattering we start from:

$$\tau_{\text{ep}}^{-1}(\mathbf{q}) = -\left(\frac{2}{N_{\mathbf{q}}^{'}K_B T}\right)\sum_{n\mathbf{k},n\mathbf{k}'}\Gamma_{n\mathbf{k},n\mathbf{k}'}(\mathbf{q})$$

$$= -\left(\frac{2N_{\mathbf{q}}A(\mathbf{q})}{N_{\mathbf{q}}^{'}K_B T}\right)\sum_{n\mathbf{k}} f_{\text{n}\mathbf{k}}\left(1 - f_{\text{n}\mathbf{k}+\mathbf{q}}\right)\delta\left(\varepsilon_{n\mathbf{k}+\mathbf{q}} - \varepsilon_{n\mathbf{k}} - \hbar\omega_{\mathbf{q}}\right) \qquad (23)$$

We can proceed as before, applying ellipsoidal band approximation for the electrons and linear-scale approximation for acoustic phonons, then changing the sum over **k** with the integral in polar coordinates; the energy delta function thus depends only on the modulus of **K**, **Q** and their angle θ, and we can exploit the same variable substitutions to obtain:

$$\sum_{nk} f_{\text{n}\mathbf{k}}\left(1 - f_{\text{n}\mathbf{k}+\mathbf{q}}\right)\delta\left(\varepsilon_{n\mathbf{k}+\mathbf{q}} - \varepsilon_{n\mathbf{k}} - \hbar\omega_{\mathbf{q}}\right) = \frac{V}{(2\pi)^2\hbar^4 q}\sum_{n}\tilde{m}_n^2\int_{\varepsilon_n^0}^{\varepsilon_n^0+W} d\varepsilon\ f_{\varepsilon}\left(1 - f_{\varepsilon+\hbar\omega_q}\right) \quad (24)$$

where:

$$\tilde{m}_n^2 = \frac{m_{nx}^* m_{ny}^* m_{nz}^*}{\sqrt{m_{nx}^* m_{ny}^* + m_{nx}^* m_{nz}^* + m_{ny}^* m_{nz}^*}} \qquad (25)$$

furthermore:

$$\frac{N_q}{N_q^{'}} = -\left(\frac{K_B T}{1 + N_q}\right) \qquad (26)$$

thus, at the end:

$$\tau_{\text{ep}}^{-1}(q) = \left(\frac{V A(q)}{2\pi^2\hbar^4 q(1 + N_q)}\right)\sum_{n}\tilde{m}_n^2\int_{\varepsilon_n^0}^{\varepsilon_n^0+W} d\varepsilon\ f_{\varepsilon}\left(1 - f_{\varepsilon+\hbar\omega_q}\right) \qquad (27)$$

$$= \left(\frac{C_{DP}}{2\pi\hbar^4} + \frac{C_{PZ}q^2}{2\pi\hbar^4\left(q^2 + q_0^2\right)^2}\right)\frac{1}{(1 + N_q)}\sum_{n}\tilde{m}_n^2\int_{\varepsilon_n^0}^{\varepsilon_n^0+W} d\varepsilon\ f_{\varepsilon}\left(1 - f_{\varepsilon+\hbar\omega_q}\right) \qquad (28)$$

For what concerns other phonon scattering processes, a simple parametrization is proposed in the seminal article of Callaway [29]:

$$\tau_{\mathrm{ph}}^{-1}(q) = \mathrm{A}\omega_q^4 + BT^3\omega_q^2 + \frac{v_s}{L} \tag{29}$$

where A describes the scattering by point impurities, B the phonon-phonon scattering including both normal (crystal momentum-conserving) and umpklapp scattering, and v_{s}/L the boundary scattering (L is a characteristic sample length; in our model L=1 mm). In the following numerical examples we will use values for A and B given in Ref. [29]. While probably not appropriate for quantitative predictions, Equation (29) is sufficient for the purpose of qualitative description of general trends.

Supplementary Note 6: Diffusive Seebeck model

The formulation of diffusive Seebeck for a multiband effective-mass model is based on the Boltzmann Transport Equation in relaxation time approximation, and band energies modeled by non-isotropic effective masses. The model is described in detail in Ref. [30] where it was first applied to LAO/STO. Here we can just recall the final expression. The DC conductivity (in 3D) in direction j is given by:

$$\sigma_j = \frac{\sqrt{2}\mathrm{e}^2}{\pi^2\hbar^3 k_B T}\sum_{n=1}^{N_b}\frac{\sqrt{m_{nx}^* m_{ny}^* m_{nz}^*}}{m_{nj}^*}\int_{\varepsilon_n^0}^{\varepsilon_n^0+W_n} d\varepsilon\,\tau(\varepsilon)\left(-\frac{\partial f}{\partial \varepsilon}\right)\left(\varepsilon-\varepsilon_n^0\right)^{3/2} \tag{30}$$

The Seebeck associated to the nth band is $S_{nj} = \Lambda_{nj}/\sigma_j$, where:

$$\Lambda_{nj} = -\frac{\sqrt{2}\,\mathrm{eN_b}}{\pi^2\hbar^3 k_B T^2}\frac{\sqrt{m_{nx}^* m_{ny}^* m_{nz}^*}}{m_{nj}^*}\int_{\varepsilon_n^0}^{\varepsilon_n^0+W_n} d\varepsilon\,\tau(\varepsilon)\left(-\frac{\partial f}{\partial \varepsilon}\right)(\varepsilon-\varepsilon_F)\left(\varepsilon-\varepsilon_n^0\right)^{3/2} \tag{31}$$

Finally, the diffusive Seebeck is obtained as:

$$S_j = \left(\frac{1}{\mathrm{N_b}}\right)\sum_{n=1}^{N_b} S_{nj} = \left(\frac{1}{\sigma_{\mathrm{j}}\mathrm{N_b}}\right)\sum_{n=1}^{N_b}\Lambda_{nj} \tag{32}$$

Notice that, although explicitly dependent on the inverse conductivity, S_j depends smoothly on the fundamental ingredients which determine the conductivity amplitude (relaxation time and electron velocities) since they equally appear in the numerator and denominator in $S_{nj} = \Lambda_{nj} / \sigma_j$. At low T the Fermi function derivative reduces to a delta function, and the integrands of Λ_{nj} and σ_j almost cancel out. It follows that in practice, it is not possible to obtain a diverging diffusive Seebeck as a consequence of the conductivity suppression. This represents an intrinsic threshold to the maximum amplitude that can be reached by diffusive Seebeck (according to the literature, this should be of the order of 10^3 μV/K). On the other hand, the S_j^{g} dependence on the inverse conductivity (Equation (21)) is real and does not cancel at low temperature, thus no such limitation exists on the attainable amplitude.

Supplementary Note 7: Electronic relaxation time

Since we are mainly interested in the very low-T regime, we can consider a minimal model for the electronic relaxation time including acoustic-phonon scattering (AP) and impurity scattering (IS). AP is treated within elastic deformation potential approach:

$$\tau_{\mathrm{AP}}^{-1}(\varepsilon) = \frac{(2\tilde{m})^{3/2} K_{\mathrm{B}} T\, D^2 \varepsilon^{1/2}}{2\pi\hbar^4 \rho v_{\mathrm{s}}^2} \tag{33}$$

where the electron energy is relative to the CBB. For IS we adopt the well known Brooks-Herring formula:

$$\tau_{\mathrm{IS}}^{-1}(\varepsilon) = \frac{\pi n_I Z^2 e^4 \varepsilon^{-3/2}}{\sqrt{2\tilde{m}}(4\pi\kappa_0\kappa)^2} \left[\log\left(1 + \frac{8\tilde{m}\varepsilon}{\hbar^2 q_0^2}\right) - \frac{1}{1 + \left(\hbar^2 q_0^2 / 8\tilde{m}\varepsilon\right)} \right] \tag{34}$$

where n_{I} is the impurity concentration, Z the impurity charge. For localized states, we assume the following hopping frequency expression:

$$\tau_{\mathrm{h}}(\varepsilon) = \tau_{\mathrm{h0}}\, \mathrm{e}^{-\left(\frac{\varepsilon_{\mathrm{CBB}}-\varepsilon}{K_B T}\right)} + \tau_{\mathrm{h0}}\, \mathrm{e}^{-\left(\frac{E_{\mathrm{T}}}{K_B T}\right)^{1/3}} \quad (35)$$

where τ_{h0} is a characteristic hopping time, and the two exponentials represent the probability of hopping by thermal excitation (for energies lower than the mobility edge $\varepsilon_{\mathrm{CBB}}$) and by tunneling across an energy barrier E_{T}, respectively. For the LAO/STO well simulation we use τ_{h0}= 10^{-11} sec, and E_{T}= 3 meV (that is in our model the energy separation between two localized states).

Supplementary Note 8: Test cases

Bulk $SrTiO_3$ n-doped (band regime) - In its simplest form, the electronic structure of n-doped bulk STO can be configured in terms of three t_{2g} degenerate conduction bands of d_{xy}, d_{xz}, d_{yz} character with strongly anisotropic effective masses: $m^*_{xy,j}$ = (0.7, 0.7, 8.8)m_{e}, $m^*_{xz,j}$ = (0.7, 8.8, 0.7)m_{e}, and $m^*_{yz,j}$ = (8.8, 0.7, 0.7)m_{e}. Results for $n_{3\mathrm{D}}$= 2x10^{19} cm^{-3} are shown in Supplementary Figure 2. In this example the electron-phonon scattering frequency τ_{ep}^{-1} (Supplementary Figure 2(A)) only includes the deformation potential (i.e. K_{em}=0). We see that τ_{ep}^{-1} grows with the phonon frequency at fixed T, and decreases at a given phonon frequency with increasing T. Its dependence on $\hbar\omega_q$ and T is dominated by two factors: the inverse Bose occupancy, and the integral over the electron-hole occupancy (see Equations (28), (29) and (30)). For what concern the τ_{ep}^{-1} dependence on the integral, in the T=0 limit the phonon can only be absorbed by electrons in the energy range $\varepsilon_n \subseteq [\varepsilon_{\mathrm{F}} - \hbar\omega_q, \varepsilon_{\mathrm{F}}]$. Thus, the number of available electronic states, and so the scattering rate, grows linearly with $\hbar\omega_q$ and saturates at $\hbar\omega_q = (\varepsilon_{\mathrm{F}} - \varepsilon_0)$ corresponding to the full occupied range $\varepsilon_n \subseteq [\varepsilon_0, \varepsilon_{\mathrm{F}}]$. With increasing T this condition is progressively relieved by the partial occupancy, saturation occurs at much higher values and it is not sharp anymore. At fixed ω_q, on the other hand, the occupancy factor $f_\varepsilon\left(1 - f_{\varepsilon+\hbar\omega_q}\right)$ decreases with increasing T.

In Supplementary Figure 2(B) we report the total phonon scattering frequency $\tau_{\text{ep}}^{-1}+\tau_{\text{ph}}^{-1}$. Since τ_{ph}^{-1} has a strong ~T^3 dependence, it becomes preponderant over electron-phonon at increasing T, causing the phonon-drag to quickly fade away with increasing T. Indeed, only at a very low temperature the profile of total scattering is visibly affected by electron-phonon. The phonon-drag T-behavior can be understood considering the relative scattering ratio (see Equation (1) and (3)):

$$S^{\text{g}} \approx \frac{N_q^{'}}{k_B T}\left(\frac{\tau_{\text{ep}}^{-1}(q)}{\tau_{\text{ph}}^{-1}(q)+\tau_{\text{ep}}^{-1}(q)}\right) \tag{36}$$

displayed in Supplementary Figure 2(C); starting from low T, we see that the ratio first quickly grows with T, reaching its maximum at about 50-60 K (~$T_{\text{D}}/10$, where T_{D}=500 K), and then it smoothly decreases. In Supplementary Figure 2(E) the phonon-drag is shown, together with diffusive and total Seebeck; consistently with Supplementary Figure 2(C), phonon-drag reaches its maximum magnitude at about 50 K and vanishes at room T. We stress that our phonon-drag modeling is only based on acoustic-phonon scattering, while at room T other electron-phonon scatterings (primarily polar optical phonon scattering) are expected to dominate. Thus albeit qualitatively reasonable, these results have the only purpose of illustrating the model characteristics.

Bulk $SrTiO_3$ n-doped (band plus localized states) - It is well known that the transport properties of STO are characterized by polaronic behavior and electron localization. Similar behavior has been also reported for LAO/STO [31]. A simple way to model these states is through poorly-dispersed mass-renormalized bands laying below the CBB, and assuming that conduction across these states can only occur by hopping. In the example shown in Supplementary Figure 3 we consider three t_{2g} conduction bands (as in the previous test-case) plus one localized state centered 5 meV below the CBB, with W=10 meV and $m^*_{\text{loc},j}$= (3.0, 3.0, 8.8)m_{e}. For the same charge density ($n_{3\text{D}}$= 2×10^{19} cm^{-3}) the electron-

phonon scattering at low phonon frequency (Supplementary Figure 3(A)) is enhanced with respect to the previous case (Supplementary Figure 2(A)) and shows a dome-like feature of width equal to *W*. The dome is progressively smoothed with increasing *T*. In the total scattering frequency (Supplementary Figure 3(A)) the electron-phonon dome is well visible up to about T=50 K, while above T=50 K it becomes more and more inessential (we use blue to red color change to emphasize the transition).

The localized state presence thus rises the phonon drag amplitude and shifts its maximum weight towards lower temperatures: phonon drag (Supplementary Figure 3(D), violet circles curve) is now peaked at about 40 K and visibly enhanced in amplitude (140 μV/K) with respect to the previous case (100 μV/K). We also monitored the phonon-drag change with charge density. For increasing density, the system behavior becomes progressively band-like and phonon-drag is reduced accordingly. The system evolution with density can be understood from resistivity (Supplementary Figure 3(C)): for n_{3D}= 2×10^{19} cm^{-3} a temperature-driven metal-insulating transition is visible, due to the presence of the localized state which at T=0 hosts all the available charge (in the previous test case, the same charge density was fully hosted by the conduction bands, and the resistivity was metallic-like). For increasing density, the zero-*T* Fermi energy progressively rises, until it is brought above the CBB, whence resistivity becomes metallic-like through the whole temperature range.

LAO/STO well - The model is built to reproduce as closely as possible the most important features of the Seebeck measured under negative voltage at T=4.2 K (we are specifically referring to the LAO/STO sample displayed in the main text): a) the distance in V_g between two consecutive oscillations (about 0.6 V); b) the charge density change corresponding to each oscillation (about 10^{10} electrons × cm^{-2}); c) the diverging values of *S*, increasing in amplitude by about 3 orders of magnitude across a V_g interval of about 14 V, with 12 oscillations interpreted as the crossing of E_F through an

equal number of polaronic levels. All these required features represent sharp constraint on the basic characteristics of the model (energies, effective masses, bandwidth), with little freedom left for alternative arrangements. Very importantly, as explained in the main text, these features in no case can be matched with the diffusive Seebeck regime, but can be only accounted by phonon-drag.

In order to reproduce the 12 oscillations displayed by the measurement on the main sample, we include in the model a series of 12 localized states, regularly distributed in energy below the CBB (parameters used for the model are listed in Supplementary Table 1; charge density and density of states of this electronic structure are displayed in Figure 3 of the main article). These localized states, separated from each other by 3 meV at the band bottom, are not identical: moving from higher to lower energies, the associated bandwidth and DOS become smaller and smaller, while the effective masses become larger, so that charge localization is progressively enhanced while E_F moves away from the CBB.

For what concern the fundamental understanding of the localized states, in literature they are typically related to two possible sources, which however do not exclude each other and can occur simultaneously: a) Anderson localization (disorder) which is known to affect the LAO/STO interface; (the presence of an Anderson tail of localized states just below the conduction edge of oxide heterostructures is well documented in literature [32]); b) Mott localization, invoked to explain other correlation phenomena observed in this system (see e.g. capacitance enhancement described in [7]). Indeed, at very low charge density, the charge can localize in Ti^{3+} sites, eventually helped by structural deformations (polarons) even in absence of structural disorder.

To mimic the experiment, the simulation assumes that at zero voltage the lowest conduction band is occupied by a mobile carrier density (n_{2D}~1.2×10^{13}cm^{-2}) typical for LAO/STO. Then, it is assumed that the leading effect of a negative V_g is the progressive charge depletion, thus mapping $S(E_F)$ is substantially equivalent to mapping $S(V_g)$. Seebeck and resistivity are then calculated as a function of the progressively decreasing E_F. Additional effects of negative V_g, such as a possible change in the

STO dielectric permittivity at the interface [26], are inessential since our simplified model only includes by construction electronic states confined in a single interface layer. We underline that specific *quantitative* features of the model (energies, effective masses, bandwidths) are set to reproduce as closely as possible the characteristics of the S measurements in terms of number of oscillations, oscillation amplitude, S vs. V_g absolute value. Of course these characteristics change, within a certain extent, from sample to sample, and so must do the parameters of the model. In particular the values in Supplementary Table I chosen for the numerical simulation are adapted to the sample shown in the main part of the article.

The resulting phonon-drag, diffusive Seebeck, and resistivity are displayed in Figure 3 of the main text. Here we add (Supplementary Figure 4) the analysis of their state-by-state contributions, which is useful to highlight some key aspects of their behavior.

Consider first the diffusive Seebeck of each individual state (Supplementary Figure 4B)): each of them displays a negative-S region followed, after crossing the S=0 axis, by a positive bell-like feature. Indeed, according to the Mott formula [33] $S_d \approx -[\partial n/\partial \varepsilon]_{\varepsilon=\varepsilon_F}$, S_d must change from negative to positive values while moving through the localized DOS, corresponding to positive and negative DOS derivatives in the regions before and after the DOS peak. This captures a general feature of diffusive Seebeck: S_d must necessarily oscillate through the zero while moving across localized DOS, where with 'localized' it is implied a series of narrow DOS, either separated by energy gaps or even partially overlapping, but with well distinct peaks (this is the case of our model). This constraint does not apply to the state-resolved S_g (Supplementary Figure 4A)) which indeed do not change sign while E_F moves across the localized states.

Notice also that for a model made of multiple overlapping bands shifted from each other in energy at the bottom, E_F would not cross any DOS peaks, and in turn S_d could display non-zero centered oscillations corresponding to the E_F crossing the onset of each band bottom. However, as extensively

discussed in the main article, in the band regime the amplitude of diffusive Seebeck at low T is totally discardable on the scale of the measured Seebeck. In our intensive attempts to simulate the experiment on the basis of a multi-band modeling, S_d could never be found to exceed a few tents of μV/K at low T, no matter how large the DOS slope (i.e. the effective masses) could be. On the basis of this analysis, we can confidently exclude that diffusive Seebeck could ever cause the huge, furiously oscillating thermopower observed in the measurements.

Finally, in Supplementary Figure 5 we report the electron-phonon scattering frequency relative to our localized state model, as a function of the phonon frequency at varying 2D doping concentration and fixed T=4.2 K. In Supplementary Figure 5(A) only the deformation potential contribution is included. The dome-like feature is similar to that seen in Supplementary Figure 3(A), but the values are much amplified (by order of magnitudes) for this extremely low-density regime. In Supplementary Figure 5(B) we show again the electron-phonon scattering frequency, but also adding the piezoelectric contribution. The latter becomes dominant in the very low frequency range, as a consequence of the $A(q) \approx q^{-1}$ behavior (Equation (22)). We see that starting from low doping (red solid curve), the scattering first increases with E_F due to the progressive increase of W and, in turn, of available electronic transitions. Then, a regime change occurs (highlighted by the red-to-blue color change) and the scattering start to decrease with E_F in consequence of the progressive decrease of the effective masses. For each curve, the phonon frequency region where the scattering is effective roughly follows the DOS profile of the electronic state crossed by E_F at that doping.

Supplementary References

---

[1] Narayan, V. et al., Density-dependent thermopower oscillations in mesoscopic two-dimensional electron gases, New J. Phys. **16,** 085009 (2014)

[2] Narayan, V. et al., Evidence of Novel Quasiparticles in a Strongly Interacting Two-Dimensional Electron System: Giant Thermopower and Metallic Behaviour, *J. Low Temp. Phys.* **171,** 626–631 (2013)

[3] Wu, J. et al., Large Thermoelectricity via Variable Range Hopping in Chemical Vapor Deposition Grown Single-layer MoS_2, *Nano Letters* **14(5),** 2730–2734 (2014)

[4] Liao, Y. C., Kopp, T., Richter, C., Rosch, A., & Mannhart J., Metal-insulator transition of the $LaAlO_3$-$SrTiO_3$ interface electron system, *Phys. Rev. B* **83,** 075402 (2011)

[5] Scheibner, R., Buhmann, H., Reuter, D., Kiselev, M.N., &Molenkamp, L.W., Thermopower of a Kondo Spin-Correlated Quantum Dot, *Phys. Rev. Lett.* **95,** 176602 (2005)

[6] Goswami, S., et al., Highly Enhanced Thermopower in Two-Dimensional Electron Systems at Millikelvin Temperatures, *Phys. Rev. Lett.* **103,** 026602 (2009)

[7] Li, L., Richter, C., Paetel, S., Kopp, T., Mannhart, J. & Ashoori, R. C., Very large capacitance enhancement in a two-dimensional electron system, *Science* **332,** 825–828 (2011)

[8] Li L., L., Richter, C., Mannhart, J., & Ashoori, R. C., Coexistence of magnetic order and two-dimensional superconductivity at $LaAlO_3/SrTiO_3$ interfaces, *Nature Phys.* **7,** 762–766 (2011)

[9] Ariando, et al., *Electronic phase separation at the $LaAlO_3/SrTiO_3$ interface, Nat. Commun.* **2,** 188–194 (2011)

[10] Sing, M. et al., Profiling the Interface Electron Gas of $LaAlO_3/SrTiO_3$ Heterostructures with Hard X-Ray Photoelectron Spectroscopy, *Phys. Rev. Lett.* **102,** 176805 (2009)

[11] Cancellieri, C. et al., Interface Fermi States of $LaAlO_3/SrTiO_3$ and Related Heterostructures, *Phys. Rev. Lett.* **110,** 137601 (2013);

[12] Cancellieri, C. et al., Doping-dependent band structure of $LaAlO_3/SrTiO_3$ interfaces by soft x-ray polarization-controlled resonant angle-resolved photoemission, *Phys. Rev. B* **89,** 121412(R) (2014)

[13] Berner, G., et al., Direct k-Space Mapping of the Electronic Structure in an Oxide-Oxide Interface, *Phys. Rev. Lett.* **110,** 247601 (2013)

[14] Breitschaft, M., et al., Two-dimensional electron liquid state at $LaAlO_3$-$SrTiO_3$ interfaces, *Phys. Rev. B* **81,** 153414 (2010)

[15] Baylin, M., Transport in Metals: Effect of the Nonequilibrium Phonons, *Phys. Rev.* **112,** 1587–1598 (1958);

[16] Baylin, M., Phonon-Drag Part of the Thermoelectric Power in Metals, *Phys. Rev.* **157,** 480 (1967)

[17] Cantrell, D. G. & Butcher, P. N., A calculation of the phonon-drag contribution to the thermopower of quasi-2D electrons coupled to 3D phonons. I. General theory, *J. Phys. C: Solid State Phys.* **20,** 1985–1992 (1987);

[18] Cantrell, D. G. & Butcher, P. N., A calculation of the phonon-drag contribution to the thermopower of quasi-2D electrons coupled to 3D phonons. II. Applications, *J. Phys. C: Solid State Phys.* **20,** 1993–2003 (1987)

[19] Smith, M. J. & Butcher, P. N., A calculation of the effect of screening on phonon drag thermopower in a Si MOSFET, *J. Phys.: Condens. Matter* **1,** 1261–1273 (1989);

[20] Smith, M. J. & Butcher, P. N., Simple models of phonon-drag in 3D and quasi-2D, *J. Phys.: Condens. Matter* **2,** 2375–2382 (1990)

[21] Peeters, F. M., Wu, X., & Devreese, J. T., Exact and approximate results for the mass of a two-dimensional polaron, *Phys. Rev. B* **37**, 933–936 (1988)

[22] Ahrens, M., Merkle, R., Rahmati, B., & Maier, J., Effective masses of electrons in n-type $SrTiO_3$ determined from low-temperature specific heat capacities, *Physica B* **393**, 239–248 (2007)

[23] Ridley, B. K., *Quantum Processes in Semiconductors,* Second Edition, Clarendon Press, Oxford, 1988

[24] Balkanski, M. & Wallis, R. F., *Semiconductor Physics and Applications*, Oxford University Press, New York, 2000

[25] Kusumoto, K., Dielectric and Piezoelectric Properties of $KNbO_3$–$NaNbO_3$–$LiNbO_3$–$SrTiO_3$ Ceramics, *Jpn. J. Appl. Phys.* **45**, 7440–7443 (2006)

[26] Rössle, M. et al., Electric-Field-Induced Polar Order and Localization of the Confined Electrons in $LaAlO_3$/$SrTiO_3$ Heterostructures, *Phys. Rev. Lett.* **110,** 136805 (2013)

[27] Fletcher, R., Tsaousidou, M., Coleridge, P.T., Feng, Y. & Wasilewski, Z.R., Electron-phonon coupling and phonon drag thermopower of a very low mobility 2DEG, *Physica E* **12,** 478–481 (2002)

[28] Ang, C., Scott, J. F., Yu, Z., Ledbetter, H., & Baptista, J. L., Dielectric and ultrasonic anomalies at 16, 37, and 65 K in $SrTiO_3$, *Phys. Rev. B* **59,** 6661–6664 (1999)

[29] Callaway, J., Model for Lattice Thermal Conductivity at Low Temperatures, *Phys. Rev.* **113**, 1046–1051 (1959)

[30] Filippetti, A. et al., Thermopower in oxide heterostructures: The importance of being multiple-band conductors, *Phys. Rev. B* **86,** 195301 (2012)

[31] Yamada, Y., Sato, H. K., Hikita, Y., Hwang, H. Y. & Kanemitsu, Y., Measurement of the Femtosecond Optical Absorption of $LaAlO_3$/$SrTiO_3$ Heterostructures: Evidence for an Extremely Slow Electron Relaxation at the Interface, *Phys. Rev. Lett.* **111**, 047403 (2013)

[32] Huang, Z., et al., Conducting channel at the $LaAlO_3$/$SrTiO_3$, interface, *Phys. Rev. B* **88,** 161107(R) (2013)

[33] Mott, N. F., *Conduction in Non-Crystalline Materials*, Oxford, Clarendon, 1987, p. 53
